# Caries incidence of the first permanent molars according to the Caries Assessment Spectrum and Treatment (CAST) index and its determinants in children: a cohort study

**DOI:** 10.1186/s12903-021-01612-1

**Published:** 2021-05-13

**Authors:** Zeinab Mahboobi, Afsaneh Pakdaman, Reza Yazdani, Leila Azadbakht, Ahmad R. Shamshiri, Azadeh Babaei

**Affiliations:** 1 Department of Community Oral Health, School of Dentistry, Tehran University of Medical Sciences, P.O. Box 1439955991, Tehran, Iran; 2 Department of Community Nutrition, School of Nutritional Sciences and Dietetics, Tehran University of Medical Sciences, Tehran, Iran; 3 Department of Community Oral Health, Research Centre for Caries Prevention, Dental Research Institute, School of Dentistry, Tehran University of Medical Sciences, Tehran, Iran; 4 Department of Community Oral Health, School of Dentistry, Alborz University of Medical Sciences, Karaj, Iran

**Keywords:** Child, Dental caries, Incidence rate

## Abstract

**Background:**

There are limited information on caries incidence, especially from developing countries, the aim of the present study was to explore caries incidence in the first permanent molar teeth according to the CAST index in 7- to 8-year-old-children and its socio-demographic, oral health related and diet determinants.

**Methods:**

A multi-stage cluster random sample of 7–8 years old children was applied in Tehran, Iran. The oral examination using the CAST index and the Oral Hygiene Index-Simplified (OHI-S) performed by trained dentists in 2017 and 2019 calibrated with an expert (Kappa of 0.89 and 0.76, respectively). A 3-day food record was used to record sugary snacks consumption. Oral health related knowledge of the parents was assessed using a valid and reliable self-administered questionnaire. The data were analyzed using the SPSS software version 23.0 and descriptive and analytical statistics including the negative binomial regression was applied.

**Results:**

Two hundred and ninety schoolchildren aged 7–8 years old were followed up for two years. All of them had complete data obtained via oral examination and questionnaires. The annual caries incidence rate was 0.16 and 53% (95% CI 47.4–58.9) of the children developed at least one new dental caries (enamel or dentine) during two years. Multi-variate analysis revealed that the children of mothers with high school education or diploma (IRR = 1.47, 95% CI 1.02–2.12; p = 0.04) and those with low socio-economic status (IRR = 1.86, 95% CI 1.27–2.73; p < 0.001) were more likely to develop caries. There was no significant association between gender, father’s educational level, child birth order, housing area per person, OHI-S score, oral health knowledge of parents, and sugary snacks consumption per day and caries increment at an individual level.

**Conclusion:**

This 2-year longitudinal study on 7- to 8-year-old children showed that caries incidence according to the CAST index was associated with socio-economic status and mother education but not associated with having 2 or more sugary snack per day and oral hygiene status.

## Background

Dental caries is one of the most common diseases, especially among children. The determinants of dental caries are well addressed in the previous studies [1]. However, there is still lack of longitudinal evidence for caries contributing factors especially in developing countries. Considering that conducting the longitudinal studies is costly and time-consuming and is therefore difficult to perform, nonetheless, it provides strong evidence to assess the long-term impact of determinants of dental caries, particularly in a specific population.

Reporting diseases using appropriate indexes is of particular importance for planning purposes. This is especially important in developing countries where there are limited resources and a wide range of dental caries from initial stages to pulp involvement [2]. Several caries detection indices have been used to report caries outcome such as DMFT/dmft, International Caries Detection and Assessment System (ICDAS) and Pulpal Involvement-Ulceration-Fistula-Abscess (PUFA/pufa). However, there is still debate on the level of detailed information needed on the severity of the disease at the individual and population levels. The Caries Assessment Spectrum and Treatment (CAST) index which was recently developed by Frencken et al. in 2011 [2] measures a wide range of oral health problems from initial caries to pulp involvement and abscess. This helps health managers to polarize resources to the most disadvantaged subjects.

The determinants of dental caries, a complex and multi-factorial disease, include diet, saliva, and microorganisms as biological risk factors that play an important role in the context of socio-demographic, behavioral, and environmental factors [1]. Several studies have found an association between diet and dental caries; however, few longitudinal studies have investigated the relationship between caries determinants and dental caries, especially in school aged children [3]. In reporting caries incidence, it is important to apply appropriate statistical tests for proper estimations [4]. Recent studies have used negative binomial and Poisson regressions considering caries increment as count data [5–7].

The prevalence of dental caries is relatively low in 12-year-old Iranian children according to the global map of the World Health Organisation (WHO) [8]. In 2016, the national oral health survey reported a mean DMFT of 1.84 ± 0.03 in 12-year-old Iranian children [9]. There is limited evidence on caries incidence especially in more detail. The prevalence of caries in 12 years old Iranian children is considered low according to the WHO report [8] but it increases with age and is high in adults [10]. So it is necessary to detect caries in the early stages as well as to identify the predictors of caries in order to focus on preventive care for more vulnerable subjects. The purpose of the present study was to evaluate the caries incidence of the first permanent molars according to the CAST index in 7- to 8 years old children in a 2-year cohort study and its socio-economic, dietary, and oral health related determinants.

## Methods

### Study design

This longitudinal study was conducted at two time intervals in 2017 (T_1_) and 2019 (T_2_). The Ethics Committee of Tehran University of Medical Sciences approved the study protocol in both time intervals (IR.TUMS.REC.1394.1730, IR.TUMS.DENTISTRY.REC.1397.149) and written informed consent was acquired from the parents/guardians of the children.

### Setting

A multi-stage clusters random sampling method, considering schools as clusters, was applied to 7–8 year-old children in 2017 (T1) and again in 2019 (T2) and a representative sample was collected from public schools of Tehran, capital city of Iran. This study was a 2-year follow-up study of a previous survey in 2017. According to the socio-economic status, Tehran was divided into affluent (1–8) and non-affluent districts (9–19) as strata. From 19 districts, six districts were randomly selected including three districts in each stratum. As schools are gender separated in Iran, four public schools (two girls’ and two boys’ schools) selected randomly from each district, in total 24 schools. After two years, in 2019, again in the same sample of schools, 307 children were re-examined. In the selected public schools, children on average spend 4–5 h and have no main meal distributed by the school officials. The sample size was calculated considering a 30% loss to follow up and sampling framework (design effect = 2) according to the method described by Norman et al. [11].

Information about the study was provided to the Ministry of Health and Medical Education and school managers to obtain permission. Then, information sheet and consent form were sent to the parents/guardians of children for oral examination and participation in the questionnaire survey. Those children with signed consent form were re-examined at school and a questionnaire on oral health knowledge and a 3-day food record were sent to parents/guardians with a return envelope. Dental caries and oral hygiene status were recorded using the CAST and OHI-S indices, respectively.

### Data measurement

#### Independent variables

A valid and reliable 9-item questionnaire [12] was used to be completed by parents in order to assess the parents’ knowledge of self and child’s oral health. The questionnaire was used to record the oral health knowledge as well as the demographic and socioeconomic characteristics of the participants including gender, education level, child birth order, number of children in the household, housing area per person, and living district.

The parents’ education level was divided into three groups as (1) illiterate, elementary school, and middle school (0–8 years of schooling), (2) high school or diploma (9–12 years of schooling) and (3) university education including associate degree, bachelor’s degree, master’s degree, and doctorate degree (> 12 years of schooling). The socio-economic status including living area per person (m^2^ per person) and district was recorded. The average living area in square meters per person (m^2^/p) [13] categorized as less than 20 m^2^ per person and equal to or more than 20 m^2^ per person [14] was used to measure the economic status of the households. Another predictor of the economic status was the residential district according to the Ministry of Education of Iran (city district) categorized as affluent and non-affluent [15]. The oral hygiene of the children was assessed using the OHI-S at baseline and follow-up by a calibrated examiner.

The questionnaire included two questions about the role of microbial plaques in caries and gum disease, two questions about the role of sugar consumption in caries, two questions about the role of oral hygiene, one question about the role of fluoride, one question about the role of mouthwashes in dental caries prevention, and one question about the role of heredity in dental caries. The answers were based on a 5-point Likert scale ranging from “strongly agree” to “strongly disagree”. The sum score of correct answers was calculated and categorized into two groups (0–8 correct answers vs. 9 correct answers).

A three-day food record together with a written instruction were sent to parents/guardians. A written note for parents was sent on how to complete the three-day food record and knowledge questionnaire. Parents were asked to record any food item children consumed as the main meal and between meal during the three consecutive days including two weekdays and one weekend. To extract the sugary items, the food items consumed by children in between meals were categorized into 12 categories as: (1) fresh fruits, (2) water, (3) dairy products, (4) nuts, (5) cake, biscuits, cookies, (6) chocolate, (7) sugar sweetened beverages (SSB), (8) sweet desserts, (9) dried fruits, (10) salty snacks, (11) food snacks (i.e. bread and vegetable/chicken burger or cheese etc.), (12) miscellaneous (items that did not fit into the other categories). Between-meal sugary snacking was summed up and the average daily intake of sugary snacks (including categories 5, 6, 7 and 8) was calculated. This was further categorized as less than two and two or more sugary snacks per day [16].

#### Outcome variable

Dental caries was recorded using the CAST index at baseline and follow-up according to the index instruction ranging from zero to nine [17]. Oral examination was conducted in the school settings under headlamp light using disposable plain dental mirrors and probes to record the CAST index for dental caries. The description of codes is as follows: code (0) as sound; code (1) as pits and/or fissures sealed with a sealant material; code (2) as restored cavities with both (in) direct restorative material; code (3) as obvious enamel changes; code (4) as discoloration related to internal dentin caries; code (5) as obvious cavity in dentine; code (6) as pulp involvements; code (7) as a swelling or sinus tract related to infectious pulp involvement; code (8) as an extracted tooth due to caries complications and code (9) as situations not matching above codes [2]. These codes were then further categorized according to a study by Baginska et al., in 2014 as “healthy”: codes 0–2, “pre-morbidity”: code 3, “morbidity”: codes 4–5, “serious morbidity”: codes 6–7, and “mortality”: code 8 [18].

Examiners (AB, ZM) were calibrated with an expert (AP) during several clinical sessions using a visual guide [17]. Oral examination performed at two time intervals. At baseline a trained and calibrated examiner (AB) collected the data (Kappa = 0.89). In the follow-up session, another calibrated examiner (ZM) performed the data collection (Kappa = 0.76). Both examiners (AB, ZM) were calibrated with each other (Kappa = 0.88). In all stages of data collection any disagreement solved with discussion.

### Statistical methods

The SPSS software version 23 (IBM Corp, Armonk, NY, USA, released 2015) was used for data analysis. Descriptive analysis was used to evaluate the characteristics of participants at both baseline and follow up study. The outcome variable (incidence) at the individual level, the caries status of the first permanent molar teeth was recorded according to the CAST code (0–9). Then, according to Baginska et al., codes 0, 1, 2 were considered as healthy “0” and other codes were considered as unhealthy “1” excluding code 9 as missing. The scores of four permanent molar teeth were summed up and the final score was calculated as the difference between the two examinations, which was used in the Generalized Estimating Equations (GEE) analysis as the outcome variable. Considering the sampling method, Generalized Estimating Equations (GEE) analysis method was chosen. As the outcome variable was a count variable and its variance and mean values were not equal (mean = 1.35, variance = 2.05) and the data did not follow the Poisson distribution, the negative binomial link function with exchangeable correlation matrix in GEE was used [19].

The association between the independent variables and the new caries development (count variable) as the incidence rate ratio (IRR) with 95% confidence interval was estimated. As for the sampling framework, the schools were considered as the *between subject variable* and children’s identification number was as the *within subject variable* in the GEE analysis. The variables with p-values of less than 0.20 in bi-variate analysis were included in multiple regression analysis and the significant level was set at 0.05.

## Results

**Fig. 1:**
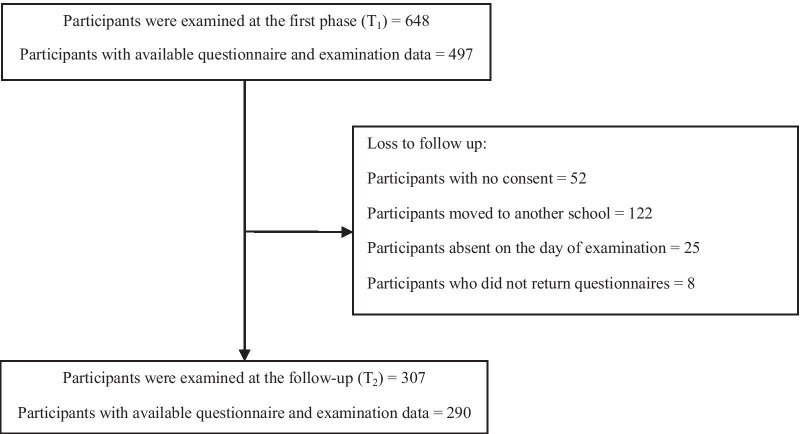
Flow diagram of the number of study subjects at both examinations as (T1) and (T2) and reasons for loss to follow-up

Of 497 children aged 7- to 8-year-old in 2017, 307 were re-examined and 290 had complete data in 2019 (response rate = 58.4%). The distribution of demographic, socioeconomic, oral hygiene status, and sugary snacks consumption per day of the participants as well as the parents’ knowledge of oral health on the first examination (T_1_) and follow-up (T_2_) and lost to follow-up are presented in Table 1. There was no significant difference in characteristics such as demographics or socio-economic status between those who participated in the follow-up and those who did not (loss to follow up). The detail of the study sampling presented in Fig. 1. Data analysis was assigned to the 290 schoolchildren who had complete data both in 2017 and 2019.

**Table 1:** Characteristics of the participants at the first phase and at 2-year follow-up

Variables	Baseline (T1) (n = 497) n (%)	Follow-up (T2) (n = 290) n (%)	Loss to follow-up (n = 207) n (%)
Gender			
Boy	214 (43.1)	116 (40.0)	98 (47.3)
Girl	283 (56.9)	174 (60.0)	109 (52.7)
Father’s education			
Illiterate/elementary school/middle school (0–8 years of schooling)	87 (17.5)	54 (18.6)	30 (14.5)
High school or diploma (9–12 years of schooling)	179 (36)	98 (33.8)	74 (35.7)
Associate/bachelor’s/master’s degree/doctorate (> 12 years of education)	231 (46.5)	138 (47.6)	103 (49.8)
Mother’s education			
Illiterate/elementary school/middle school (0–8 years of schooling)	68 (13.7)	41 (14.1)	25 (12.1)
High school or diploma (9–12 years of schooling)	213 (42.8)	131 (45.2)	76 (36.7)
Associate/bachelor’s/master’s degree/doctorate (> 12 years of education)	216 (43.5)	118 (40.7)	106 (51.2)
Child birth order			
First	297 (59.8)	169 (58.3)	127 (61.4)
Second	168 (33.8)	94 (32.4)	75 (36.2)
Third	30 (6.0)	26 (9.0)	4 (1.9)
Forth and more	2 (0.4)	1 (0.3)	1(.5)
Number of children in the household			
One child	164 (33.0)	73 (25.1)	75 (36.2)
Two children	278 (55.9)	162 (55.9)	119 (57.5)
Three or more children	55 (11.1)	55 (19.0)	13 (6.3)
Housing area			
m^2^/p less than 20	198 (39.5)	123 (42.4)	81 (39.1)
m^2^/p equal or more than 20	299 (60.2)	167 (57.6)	126 (60.9)
Living district			
Non affluent	270 (54.3)	178 (61.4)	92 (44.4)
Affluent	227 (45.7)	112 (38.6)	115 (55.6)
Oral health knowledge (9 questions)			
9 correct answers	105 (21.1)	51 (17.6)	43 (20.8)
0–8 correct answers	392 (78.9)	239 (82.4)	164 (79.2)
Sugary snack intake per day			
Less than two sugary snack/day		179 (61.7)	
Two or more than two sugary snack/day		111 (38.3)	
OHI-S			
Mean OHI-S	0.72 ± 0.40	1.07 ± 0.41	0.69 ± 0.38

### Demographic characteristic of study samples

In the follow-up study, most of the children were girls (60%), which was similar to the baseline examination, and the rest were boys. Regarding parents’ education level, in the baseline examination 46.5% of children had fathers with the university education, which was similar to the follow-up (47.6%). Less than half of the children had mothers with an academic degree at baseline (43.5) and follow up (40.7). More than half of the participants at both time intervals lived in non-affluent districts (54% and 61%, respectively). About 20% of the parents answered correctly to the knowledge questionnaire (9 Qs) in both surveys. In the follow-up study, about 38% of the children consumed two or more than two sugary snacks per day.

### Clinical examination

**Fig. 2:**
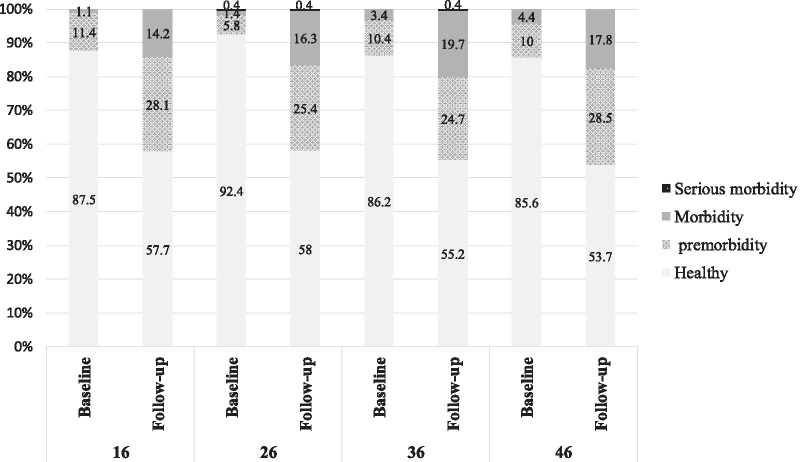
Frequency distribution of caries status according to the Baginska classification in the first permanent molars in baseline and after 2 years’ follow-up

The percentage of children with healthy first permanent molars (upper and lower) at T1 and T2 was 75.7% and 36.3%, respectively. At baseline almost all 1^st^ permanent molar teeth were healthy (80–90%) and after 2 years nearly half of teeth were healthy. At follow-up examination, about a quarter of teeth had enamel caries (pre-morbidity) and about 10–20% had dentinal lesion (Morbidity). The oral hygiene status measured using the mean (SD) of OHI-S score at baseline and follow up was 0.72 ± 0.40 and 1.07 ± 0.41, respectively. The caries status of the first permanent molars was assessed using the CAST index and grouped according to the Baginska et al. [18]. The annual incidence rate was 0.16 and 53% (95% CI 47.36–58.85) of the children developed at least one new dental caries (enamel or dentine) during two years. The caries status at baseline (T1) and follow-up (T2) is presented in Fig. 2.

The result of the bi-variate analysis showed that there was no significant association between caries incidence and gender of children (p = 0.77), oral health related knowledge of parents (p = 0.09), sugary snack consumption (p = 0.80) and oral hygiene status (p = 0.90). Multivariate analysis showed that there was a significant association between the number of decayed teeth and the education level of parents in addition to the economic status. Those participants who lived in the non-affluent districts and those children whose mothers had high school/diploma education (no university degree) were at higher risk of developing new caries [IRR: 1.86 (95% CI 1.27–2.73, p < 0.001) and IRR: 1.47 (95% CI 1.02–2.12, p = 0.04), respectively. The results of bivariate and multivariate analysis of the impact of socio-demographic and oral health related characteristics of participants and new caries development using negative binomial regression are presented in Table 2.

**Table 2:** Bivariate and multi-variate analysis of new caries development and socio-demographic variables including oral health related knowledge, snacking pattern and OHI-S according to the negative binomial regression analysis

	n (%)	IRR	p value	IRR	p value
Gender					
Boy	109 (40.2)	1.06 (0.70–1.61)	0.77		
Girl	162 (59.8)	1			
Father’s education					
Illiterate/elementary school/middle school (0–8 years of education)	50 (18.5)	1.33 (0.91–1.95)	0.14		
High school or diploma (9–12 years of education)	90 (33.2)	1.15 (0.82–1.61)	0.41		
Associate/bachelor/master of science/doctorate (> 12 years of education)	131 (48.3)	1			
Mother’s education					
Illiterate/elementary school/middle school (0–8 years of education)	38 (14)	1.34 (0.87–2.06)	0.19	1.22 (0.79–1.90)	0.38
High school or diploma (9–12 years of education)	122 (45)	1.58 (1.11–2.52)	0.01	1.47 (1.02–2.12)	0.04
Associate/bachelor/master of science/doctorate (> 12 years of education)	111(41)	1		1	
Child birth order					
2nd or more	114 (42.1)	1.04 (0.83–1.31)	0.74		
1st child or single	157 (57.9)	1			
Housing area (per person)					
< 20 m^2^	117 (43.2)	0.93 (0.71–1.21)	0.58		
≥ 20 m^2^	154 (56.8)	1			
Living district					
Non-affluent	168 (62)	2.06 (1.42–2.99)	< 0.001	1.86 (1.27–2.73)	< 0.001
Affluent	103 (38)	1		1	
Oral health knowledge					
0–8 correct answers	227 (83.8)	1.42 (0.95–2.13)	0.09		
9 correct answers	44 (16.2)	1			
Sugary snack intake per day					
Two or more than two sugary snack/day	103 (38)	0.96 (0.73–1.27)	0.80		
Less than two sugary snack/day	168 (62)	1			
OHI-S					
OHI-S	271 (100)	0.98 (0.72–1.33)	0.90		

*IRR* incidence rate ratio

## Discussion

This study evaluated the 2-year incidence of new caries in the first permanent molar teeth of 7–8 year-old children according to the CAST index and its risk factors. In the two-year follow-up, more than half of the children developed new caries in permanent first molar teeth (incidence) considering enamel and dentine lesions. The children with a low socio-economic status and those whose mothers had high school/diploma education (no university degree) developed a significantly larger number of new caries compared to the comparison group. There was no significant association between new caries development and parents' oral health related knowledge, number of children in the household, child’s birth order, gender, and frequency of sugary snacks per day and oral hygiene status of children according to OHI-S index.

Studies reporting caries incidence have reported either the incidence rate ratio (IRR) or the incidence rate per person-time at risk. A review study by Hummel et al. in 2019 found that at the general public level, the *caries incidence rate* is a promising statistic for predicting future caries increments. In this study, a meta-analysis of 32 studies including children and adolescents up to 21 years of age showed that a pooled caries incidence rate of 0.11 (0.09–0.13) per person-year at risk. In this review, considering D3 lesion showed that the annual increment ranged from 0.06 to 0.73 for DMFT. This meta-analysis showed that the pooled caries incidence rate and increment of DMFS and DMFT indexes were influenced by the method of individual studies [4].

The results of our study confirmed the findings of other studies on the impact of the socio-economic status on oral health. In our study, living district and mother’s education had a significant association with increase in number of dental caries. Most of the children with new caries development were from non-affluent districts or their mothers had high school education or diploma. This finding is in line with the results of a longitudinal study of 12-year-old children by Oritz et al., with two years of follow-up that showed a higher incidence of caries in children was associated with low socio-economic background [7].

The recent review study by Schwendicke et al. [20] reported that social position defined as parental educational/occupation or income has impact on oral health. Those with low level of social position have greater odds of having any caries lesions or caries experience [20]. It might be inferred that the impact of socio-economic status on caries development might be related to the multifactorial nature of tooth decay. Parent of children from low socio-economic status usually have other priorities rather health that make them more vulnerable. One potential reason for this finding could be having predisposing risk factors as children with low socio-economic status may have inappropriate diet or limited access to health care services.

The present study showed that the mother’s education was associated with new caries development in children. Children whose mothers had high school education or diploma had more new caries compared with the children of mothers with an academic education. Our finding was in line with the results of an Iranian survey study conducted by Ghaseminpoor et al., also found that the mother’s education level was negatively related to permanent teeth caries in 12-year-old children [9]. Also, the previous national survey on Iranian children in 2004 reported by Bayat-Movahed et al., showed that there is a significant relationship between caries and province, city of residence, family income, and parents' education level [21].Moreover, Edasseri et al., studied children aged 8–10 years with a 2-year follow-up in 2017 and found that the high education level (more than high school education) of one or both parents has a protective role against caries incidence [6]. However, longitudinal studies conducted by Peres et al., in children aged 6–18 years and Llena et al., in 10-year-olds showed that the educational level of mothers was not associated with caries development [22, 23].

We found no association between new caries development and gender, father’s education, number of children in the household, oral health related knowledge of parents, sugary snack consumption and oral hygiene status of children according to the OHI-S index. A longitudinal study of children aged 9–11 years with a three-year follow-up period conducted by Melo et al., in Brazil found that the incidence of caries at the occlusal surface of the first permanent molars was significantly associated with previous caries history and plaque accumulation on this surface. However, there was no significant relationship between the incidence of caries and the child's gender, family income level, and mother's education [24].

There was no association between two or more sugary snack consumption and caries incidence measured by CAST index in the present study. Llena et al., conducted a five-year retrospective cohort on 10-year old children and found that the predictors of caries development in the first permanent molars were consumption of sugary drinks, frequency of brushing, caries history in deciduous and molar incisor hypomineralization [22]. On the other hand, the results of a systematic review by Moynihan and Kelly in 2014 showed a relationship between caries prevalence and free sugars intake more than 10% of energy intake [25].

Our study findings was consistent with the results of a 4-year follow-up study of Chankanka et al. in 2011 reported no association between caries incidence and sugary food or drinks consumption [26]. In our study sugar consumption was not measured according to some detail indices such as the energy intake and therefore no relationship could be found. Besides, there may be under-reporting of sugary snack consumption in our study as this is unacceptable due to wide health messages on the negative impact of having too much sugar on general health. Moreover, the delivering of topical fluoride at the community level including fluoride toothpaste and twice yearly application of fluoride varnish may influence the impact of sugar consumption on caries development.

There was no association between OHI-S as a measure of oral hygiene and new caries development in the present study. No relationship was found between OHI-S and DMFT in the study of 11–14 years children by Rehman et al. [27] whereas studies conducted by Oyedele et al., on 8–14 year-old children showed that poor oral hygiene was associated with a higher chance of developing caries [28].

The results of the present study showed no relationship between oral health knowledge of parents and caries incidence, which is in line with the result of studies by Maharani et al., and Saied-Moallemi et al., also Babaie et al., that found no association between parents’ oral health related knowledge and caries prevalence in children using DMFT and CAST index, respectively [12, 14, 29].

In the present study, gender was not related to new caries development, which is in line with the results of longitudinal studies by Oritz et al., Edasseri et al. and Peres et al. [6, 7]. Moreover, being the only child or the first child in the family did not increase caries increment, which was consistent with the findings of the study by Al-Meedani et al., that found no association between caries prevalence and birth order [30]. However, Floyan et al., and Oyedele et al., found that having a sibling was associated with caries prevalence [28, 31].

Our study has some strength in terms of study design and measurement. The present study is the first study in this field reporting a more detailed index of caries including enamel and dentine lesions. It is also important that a limited number of children experienced severe complications of dental caries including swelling or sinus tract related to infectious pulp involvement and extracted teeth due to caries in the permanent first molars, which confirms a mean (SE) DMFT of 1.84 (0.03) in 12-year-old children previous reported by the Ministry of Health and Medical Education [9]. As a representative sample across the capital city was used, provide a picture on caries incidence of the permanent dentition in children.

In our study, the impact of possible risk factors and risk indicators on the caries incidence of the permanent molars were assessed including oral hygiene status and snacking pattern. Other main risk factors including topical and systemic fluoride intake and saliva characteristics were not assessed directly. In our study, all children had access to public water in Tehran containing about 0.3–0.6 ppm fluoride [32]. Fluoridated toothpaste is available in the local market and fluoride varnish delivered by the Ministry of Health and Medical Education semi-annually.

Considering the multi-factorial nature of caries, possible risk indicators were considered including the socio-economic status, parents' education, number of children in the household and child birth order. In our study, previous caries was not considered as predisposing factor. As, those risk factors and risk indicators that related to previous dental caries experience, are highly correlated with present status and considering the “co-linearity” problem of independent variables we did not entered it in our final data analysis.

However, this study had some limitations. In our study, the presence of caries may be reported higher than studies that use the DMFT index reporting cavitated dentine lesions. Therefore the risk factors affecting caries should be interpreted with caution [33]. The snacking pattern of the children was assessed using a three-day food record, which has some limitations. There might be under-reporting of dietary intake of sugary items as the limitation of this tool was previously discussed by Thompson [34]. It is recommended that two tools be used at the same time to increase the validity. Another weakness of this study was loss to follow-up. According to the design of the education system in Iran, schools may have different campuses for year 4–6. Therefore, some children move to other schools at higher grades. Our study as a longitudinal study on a representative sample of children using a comprehensive tool for caries assessment in children provides valuable information. As conducting longitudinal studies in school-aged children is challenging since these studies are expensive, time-consuming, and prone to attrition.

## Conclusion

In conclusion, the incidence of caries in the first permanent molars as measured by any increase in the number of caries from healthy to pre-morbidity, morbidity, serious morbidity and mortality stages was associated with low socio-economic status and having no academic education of mothers. However, oral hygiene status as measured according to OHI-S index and snacking was not associated with new caries development in permanent first molars. Accordingly, it is recommended that oral health promotion programs targeting children from low socio-economic status and those with lower level of mother education be planned in future studies.

## Data Availability

Data and materials are available upon request and on sensible demand from corresponding author.

## Abbreviations

CAST: Caries Assessment Spectrum and Treatment
OHI-S: Oral Hygiene Index-Simplified
IRR: Incidence Rate Ratio
DMFT: Decayed, Missed, Filled Tooth
dmft: decayed, missed, filled tooth (in deciduous system)
PUFA: Pulpal involvement, Ulceration, Fistula, Abscess
ICDAS: International Caries Detection and Assessment System
WHO: World Health Organisation
SSB: Sugar sweetened beverages
SPSS: Statistical Package for the Social Sciences
IBM: International Business Machines Corporation
GEE: Generalized estimating equation
m^2^/p: Square meters per person
SD: Standard deviation
CI: Confidence interval
